# Bessel Beam Illumination Reduces Random and Systematic Errors in Quantitative Functional Studies Using Light-Sheet Microscopy

**DOI:** 10.3389/fncel.2018.00315

**Published:** 2018-09-20

**Authors:** M. Caroline Müllenbroich, Lapo Turrini, Ludovico Silvestri, Tommaso Alterini, Ali Gheisari, Natascia Tiso, Francesco Vanzi, Leonardo Sacconi, Francesco S. Pavone

**Affiliations:** ^1^National Institute of Optics, National Research Council, Sesto Fiorentino, Italy; ^2^European Laboratory for Non-linear Spectroscopy, LENS, Sesto Fiorentino, Italy; ^3^Department of Biology, University of Padova, Padua, Italy; ^4^Department of Biology, University of Florence, Sesto Fiorentino, Italy; ^5^Department of Physics and Astronomy, University of Florence, Sesto Fiorentino, Italy

**Keywords:** spontaneous activity, zebrafish, principle component analysis, light-sheet microscopy, functional imaging, Bessel beams, flickering artifacts, striping

## Abstract

Light-sheet microscopy (LSM), in combination with intrinsically transparent zebrafish larvae, is a method of choice to observe brain function with high frame rates at cellular resolution. Inherently to LSM, however, residual opaque objects cause stripe artifacts, which obscure features of interest and, during functional imaging, modulate fluorescence variations related to neuronal activity. Here, we report how Bessel beams reduce streaking artifacts and produce high-fidelity quantitative data demonstrating a fivefold increase in sensitivity to calcium transients and a 20-fold increase in accuracy in the detection of activity correlations in functional imaging. Furthermore, using principal component analysis, we show that measurements obtained with Bessel beams are clean enough to reveal in one-shot experiments correlations that can not be averaged over trials after stimuli as is the case when studying spontaneous activity. Our results not only demonstrate the contamination of data by systematic and random errors through conventional Gaussian illumination and but,furthermore, quantify the increase in fidelity of such data when using Bessel beams.

## 1. Introduction

Light-sheet microscopy (LSM) in combination with intrinsically transparent samples like zebrafish larvae, is a choice method to elucidated neuronal activity on a organ-wide scale at cellular resolution and has yielded fast volumetric calcium activation maps over the entire encephalon (Vladimirov et al., 2014). In LSM Siedentopf and Zsigmondy (1902), a technique which affords intrinsic optical sectioning, fast acquisition rates and low photobleaching, fluorescence is excited in a thin sheet of excitation light that coincides with the focal plane of a perpendicularly placed detection objective (Huisken et al., 2004).

This very nature of uncoupled, perpendicular optical pathways for fluorescence excitation and detection entails, however, a set of drawbacks unique to LSM in the form of dark shadows that appear whenever the fluorescence-exciting light sheet is interrupted by scattering or absorbing obstacles. These refractive heterogeneities, always present to some extent even in extremely well clarified or intrinsically transparent samples, have been shown to lead to a loss of spatial resolution and a concomitant degradation in sensitivity and contrast (Chen et al., 2016). At best, dark shadowing severely affects image homogeneity, at worst, it completely obscures any feature of interest in the affected area. Considering the increasingly large dataset sizes now routinely produced in high-throughput light-sheet microscopy, high demands are placed on the automated tools to count, trace or segment the fluorescent features of interest. Consequently, background uniformity and indeed high-fidelity imaging are paramount to facilitate the extraction of meaningful insights from terabytes of data.

Furthermore, if the obstacles that obstruct the light sheet are not static, their shadows dynamically modulate the fluorescence intensity, causing an artifact here termed “flickering.” For example, hemodynamic absorption (Ma et al., 2016) of the light sheet can be particularly problematic for *in-vivo* Ca2+-imaging where the variation of fluorescence signal over time quantifies neuronal activity. We hypothesize that artifacts due to streaky shadows are non-negligible and cause loss or corruption of data obtained in light-sheet microscopy experiments. This premise is supported by a previous functional study in zebrafish which had to manually exclude neurons affected by severe flickering from further flickering from further analysis (Panier et al., 2013). We argue that dynamic flickering constitutes an inherent source of artifact potentially falsifying in light-sheet microscopy experiments and therefore any theory inferred from such observations might have to be questioned.

Aiming for an optical solution to streaking artifacts, here, we apply Bessel beams (Durnin et al., 1987, see Supplementary Materials for further information), to LSM to functional imaging of zebrafish larvae for high-fidelity interrogation of their neuronal activity.

Bessel beams have been previously applied to microscopy to extend the useful field of view or depth of field (Lorenser et al., 2014; Lu et al., 2017) or to image in scattering media either to study the scattering properties of the sample or the self-reconstructing properties of the Bessel beam itself (Fahrbach et al., 2010, 2013a). Since a Bessel beam's central core can be extremely narrow without being subject to diffraction (McGloin and Dholakia, 2005), Bessel beams have also been applied to LSM to obtain isotropic resolution (Planchon et al., 2011; Gao et al., 2014) or to increase spatio-temporal resolution (Chen et al., 2014, however mostly demonstrating results from cell cultures or *C. elegans* at a very early developmental stage. Finally, two groups have published technological development concerning Bessel beams applied to LSM (Zhang et al., 2014; Zhao et al., 2016).

Here, we report on the application of Bessel beams to functional imaging in zebrafish larva in LSM, and more specifically to the study of spontaneous activity. The investigation of spontaneous activity, by definition, implies measurements in complete absence of stimuli and therefore precludes the possibility to clean up data by averaging of repeated trials. Using a direct comparative analysis between Bessel and Gaussian illumination we first provide supporting evidence and quantification of artifacts introduced by Gaussian illumination. Further, we demonstrate that Bessel beams provide the superior accuracy and sensitivity needed to reveal strong correlations in spontaneous activity in one-shot measurements otherwise lost when using conventional Gaussian illumination.

## 2. Results

### Light-sheet microscope for *in-vivo* imaging

**Figure 1:**
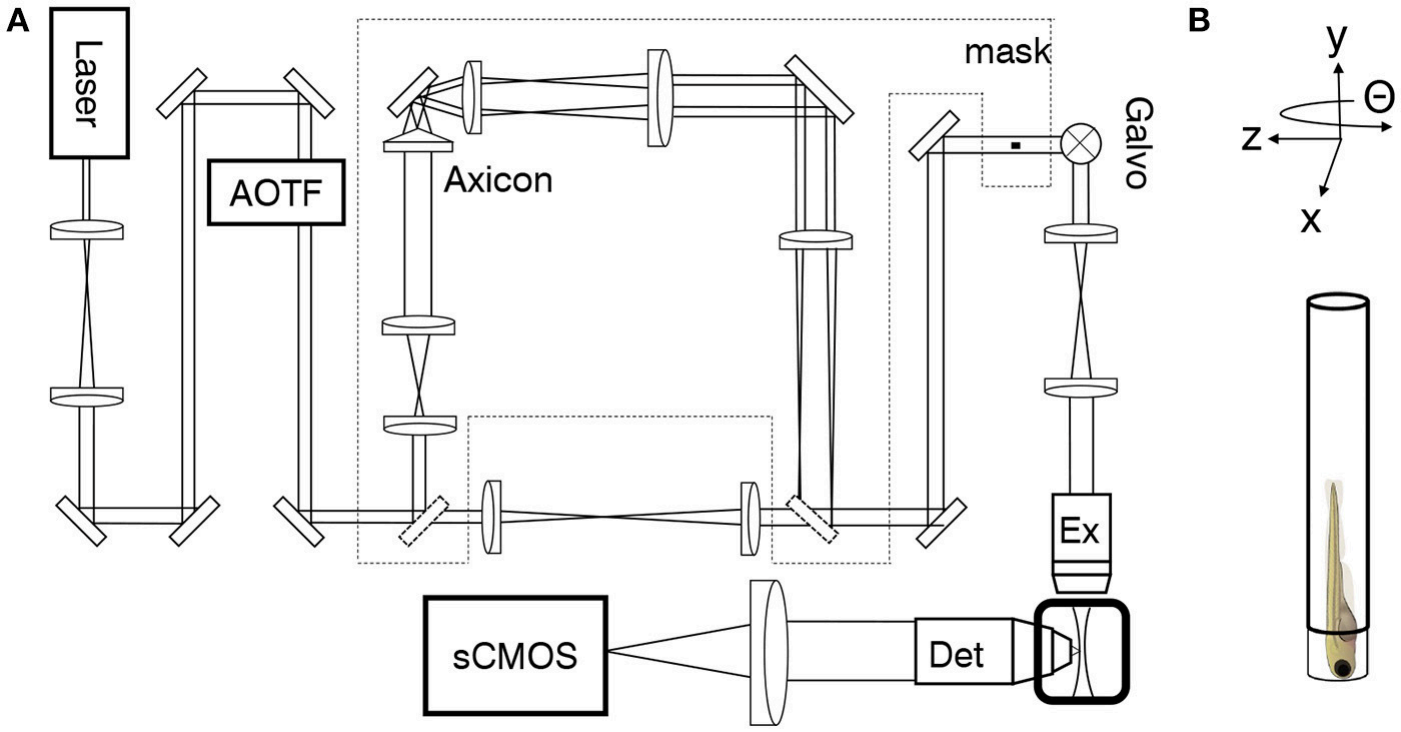
Light-sheet microscope for *in-vivo* imaging. **(A)** As an alternative to standard Gaussian illumination, a Bessel beam created by an axicon could be used. **(B)** The larva was mounted vertically on an x−, y−, z−, Θ-stage.

A custom-made light-sheet microscope (Figure 1A and Figure S2), specifically designed for *in-vivo* imaging of zebrafish larva, was used for all functional imaging experiment. In brief, a 488 nm continuous-wave diode-pump solid state laser (Excelsior, Spectra Physics, Santa Clara, USA) with 50 mW output power was used as light source and an acousto-optical tunable filter (AOTF) was used as a shutter and power regulator. Two different illumination paths could be selected by the use of flip mirrors. The first illumination path, expands the output of the laser onto a galvanometric mirror (galvo, 6220H, Cambridge Technology, Bedford, USA). The galvo is re-imaged with a 4f telescope onto the back aperture of the excitation objective (4x, 0.13 NA, air immersion, Olympus, Tokyo, Japan). The excitation objective focused the illumination beam into the sample chamber and by applying a sawtooth waveform to the galvo the beam was rapidly scanned to form a virtual light sheet which coincided with the focal plane of a perpendicularly placed detection objective (20x, 1 NA, water immersion, Olympus, Tokyo, Japan). The sample was mounted on an x−, y−, z−, Θ-stage (M-122.2DD and M-116.DG, Physik Instrumente, Karlsruhe, Germany) which allowed its precise positioning and the acquisition of z-stacks. Fluorescence was detected in a wide-field scheme with a tube lens forming an image onto the chip of a sCMOS camera (OrcaFlash4.0, Hamamatsu, Hamamatsu city, Japan). Appropriate filters are used to bandpass filter the fluorescence and reject excitation light. The camera was operated in rolling shutter mode thereby creating a virtual confocal slit. In the second illumination path a telescope adjusted the beam diameter of the Gaussian beam before the axicon (AX251-A, α = 1°, Thorlabs, Newton, USA) to minimize the Gaussian contribution produced by the round tip. The self-reconstruction length of the resulting Bessel beam (see also Figure S1) was then re-imaged with 3 additional lenses, each placed at 2f from each other respectively, into the common Gaussian light path. A circular spatial filter was used close to a Fourier plane near the galvo to filter out any residual Gaussian contribution to the Bessel beam. A heating system kept the sample chamber at a constant temperature of 28.5 °C. The microscope was controlled via custom software written in LabVIEW 2012 (National Instruments, Austin, USA) using the Murmex library (Distrio, Amsterdam, The Netherlands).

### Streaking artifacts obscure microscopic features of interest

**Figure 2:**
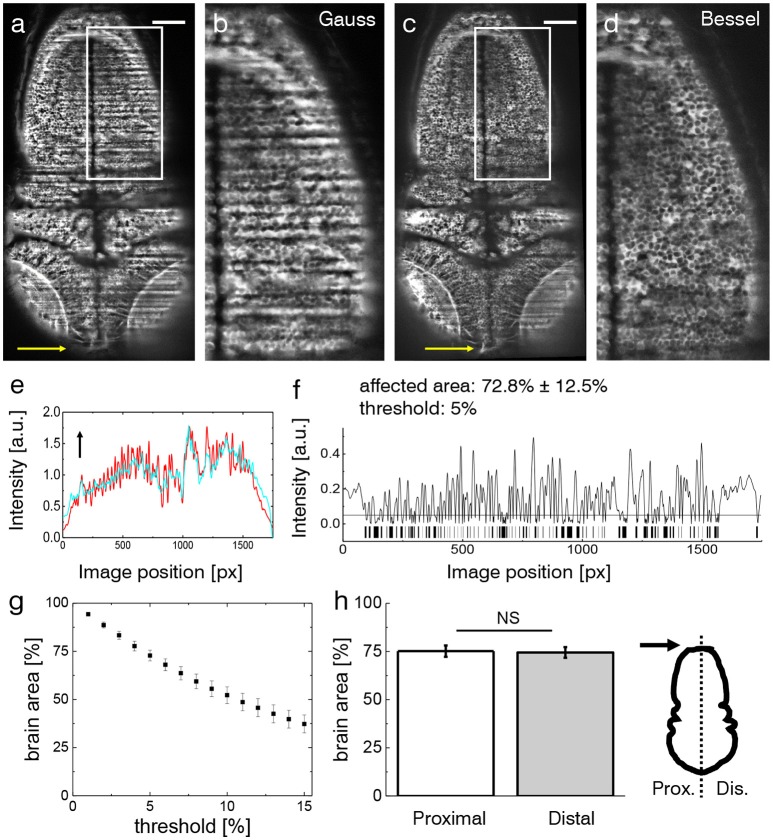
Shadow artifacts in zebrafish imaging. **(a,c)** Static shadows in the encephalon of a 3 dpf Tg(elavl3:GCaMP6s) zebrafish with cytoplasmatic expression of GCaMP imaged with Gaussian and Bessel beam illumination. Yellow arrow indicates light-sheet propagation. Scale bar: 100 μm. **(b,d)** Details showing the hindbrain. The contrast in both images has been enhanced over the entire image using Contrast-Limited Adaptive Histogram Equalization (CLAHE) in ImageJ for better visualization. **(e)** Normalized line profile averaged over the entire width of the image for Gaussian (red) and Bessel beam illumination (cyan) evidences the shadows as drops in the red curve. Arrow indicates light-sheet propagation. **(f)** Absolute difference of the line profiles in **(e)** and the resulting bar code when applying a threshold of 5 %. With this threshold 72.8 ± 12.5 % (error is standard deviation, *n* = 20 planes throughout the encephalon in *N* = 11 larvae) was affected by streaking artifacts. **(g)** Sensitivity of the affected area on the threshold. Each point is the average of *n* = 20 planes in *N* = 11 larvae, error is standard error of the mean (sem). **(h)** The difference between the half of the larva proximal and distal to the light-sheet source is not statistically significant (*p* = 0.6467, paired *t*-test of *n* = 20 planes in *N* = 11 larvae, error is sem).

The effects of streaking artifacts are visualized in Figure 2. All images were acquired using a custom-built light-sheet microscope (Figure 1) which provided cellular resolution over the entire encephalon within one field of view. The image obtained using Gaussian illumination (Figure 2a) shows degraded image homogeneity and dark shadows obscuring microscopic anatomical features of interest which are further illustrated in an inset detailing the hindbrain (Figure 2b). Notably those same features remain clearly visible when using Bessel beam illumination (Figures 2c,d). In order to quantify the extent of the area affected by strong striping, we calculated the normalized line profiles obtained over the entire width (along the illumination direction) of the image for Gauss and Bessel illumination respectively (Figure 2e) and further binarised their absolute difference (Figure 2f) with respect to a user-selected threshold to obtain a pattern similar to a bar code. By superimposing this bar pattern to the original image the percentage of 2D area affected by streaking inhomogeneity was estimated (Müllenbroich et al., 2018)). Using a threshold of I5%, we estimated that 72.8 ± 12.5% (error is standard deviation, *n* = 20 planes throughout the encephalon, *N* = 11 larvae) of the volume was affected by streaking.

The sensitivity of the area percentage on the threshold is reported in Figure 2g (using the same sample size, error is sem). The percentage area when considering only the proximal or distal half of the zebrafish respectively shows no statistically relevant difference (Figure 2h, *p*=0.6467, paired *t*-test of *n* = 20 planes in *N* = 11 larvae aged 4–5 dpf, error is sem) highlighting the limitation of double-sided illumination.

### Dynamic flickering modifies functional traces

**Figure 3:**
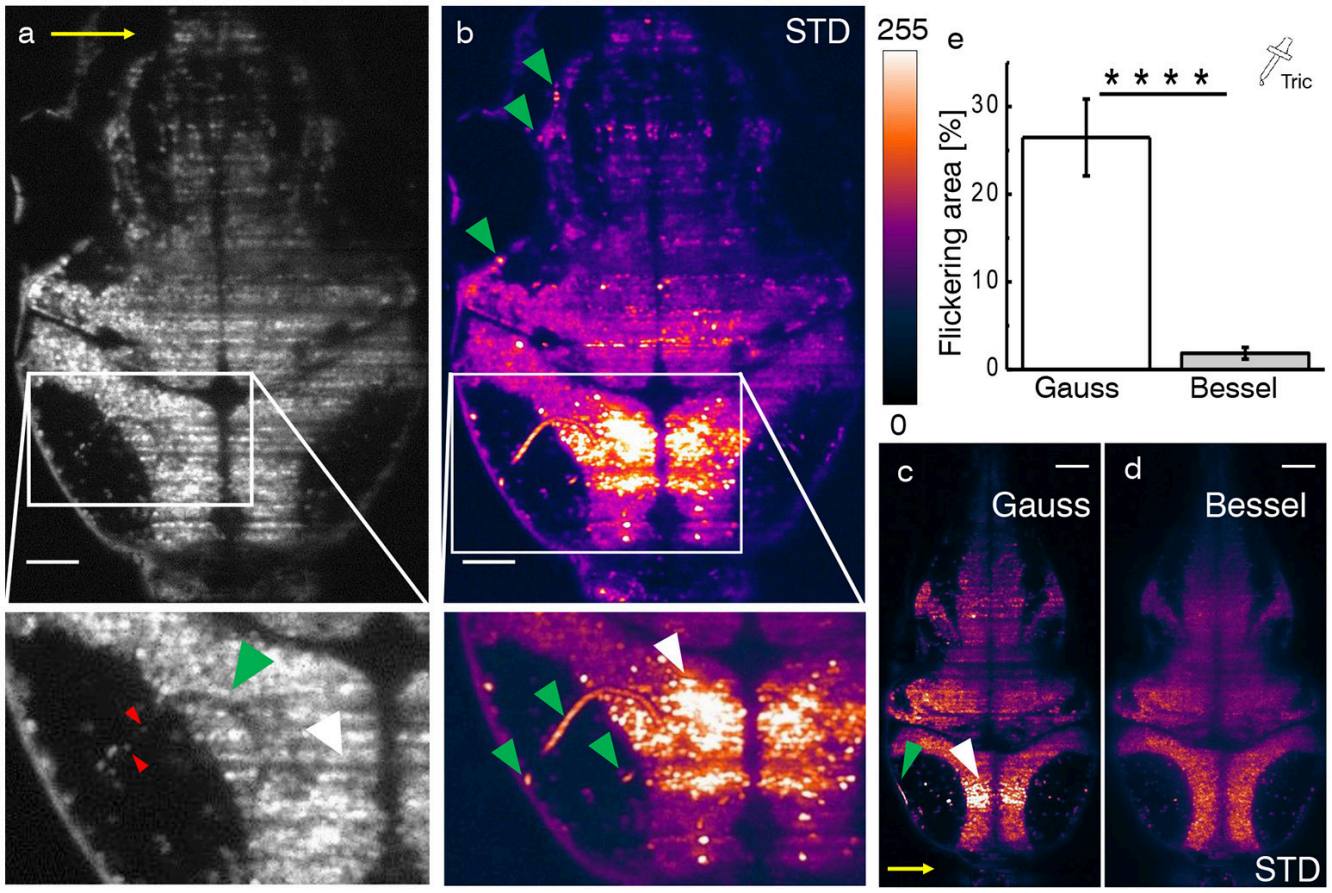
Hemodynamic flickering in zebrafish imaging. **(a)** Transverse plane of a 4dpf Zebrafish larva [Tg(elavl3:H2B-GCaMP6s)]. Red blood cells (red arrow heads) passing through vasculature (green arrow head) dynamically absorb or scatter the excitation light sheet (yellow arrow) and create areas of strongly fluctuating shadow artifacts (white arrow head). Scale bar: 50 μm. **(b)** Projection of the standard deviation over ≈ 100 ms of a time lapse recording on the plane shown in **(a)**. Each of the three segments of vasculature (green arrow heads, inset) causes a corresponding area of high standard deviation of the fluorescence intensity (white arrow head). A look-up table has been applied for clarity ranging from 0 (black) to 255 (white) for the 8-bit gray pixel value. **(c,d)** Displayed on the same brightness scale, corresponding flickering is much reduced with Bessel beam illumination. **(e)** Quantification of 2D area strongly affected by flickering for Gaussian (23.8 ± 6.5 %) and and Bessel beam illumination (0.8 ± 0.5 %) (****p ≤ 0.0001, paired *t*-test of *n* = 18 planes in *N* = 10 larvae aged 4–5 dpf, error is sem). Tricaine (160 mg l-1) was added to the fish water.

In the following, we consider functional imaging in zebrafish where neuronal activity is quantified by a relative change in fluorescence of the calcium indicator with respect to a baseline value. Additionally to previously discussed static shadows also present in intrinsically transparent zebrafish larva, dynamic shadowing caused by the movement of red blood cells leads to a fluctuation of this baseline, here termed flickering. As early as 3 days post fertilization (dpf), fully formed blood vessels (Figures 3a–c, green arrow heads) can be easily distinguished by the flow of individual blood cells (red arrow heads). Hemodynamic absorption and scattering represents a multiplicative noise source (Ma et al., 2016) and therefore has the potential to modulate significantly the sensitivity to fluorescence variations related to neuronal activity in adjacent neurons (white arrow heads). A projection of the standard deviation for ≈ 100 ms of the trace (Figure 3b) clearly evidences blood vessels by superimposing the trajectories of blood cells and further highlights the strong variations in gray values in corresponding adjacent regions (see Videos S1–S4). Notably, when displayed on the same brightness scale, the corresponding areas are free of strong flickering when using Bessel beam illumination (Figures 3c,d).

In this section, we evaluate to which extent flickering potentially masks or even falsifies neuronal activity when using Gaussian illumination. Furthermore we directly compare each quantification with its Bessel beam counterpart. Based on an approach employing standard deviation projections, we devised an automated methodology detailed in the methods 14 and Figures S3, S4. Tricaine, a general anesthetic that blocks voltage sensitive Na+ channels preferentially in neurons, was added to the fish water. The 2D area fraction affected by strong flickering was quantified at 23.8 ± 6.5% for Gaussian and 0.8 ± 0.5% for Bessel beam illumination respectively (Figure 3e, error is sem, *p* < 0.0001, paired *t*-test, *n* = 18 planes in *N* = 10 larvae aged 4–5 dpf).

#### Hemodynamic baseline contamination

**Figure 4:**
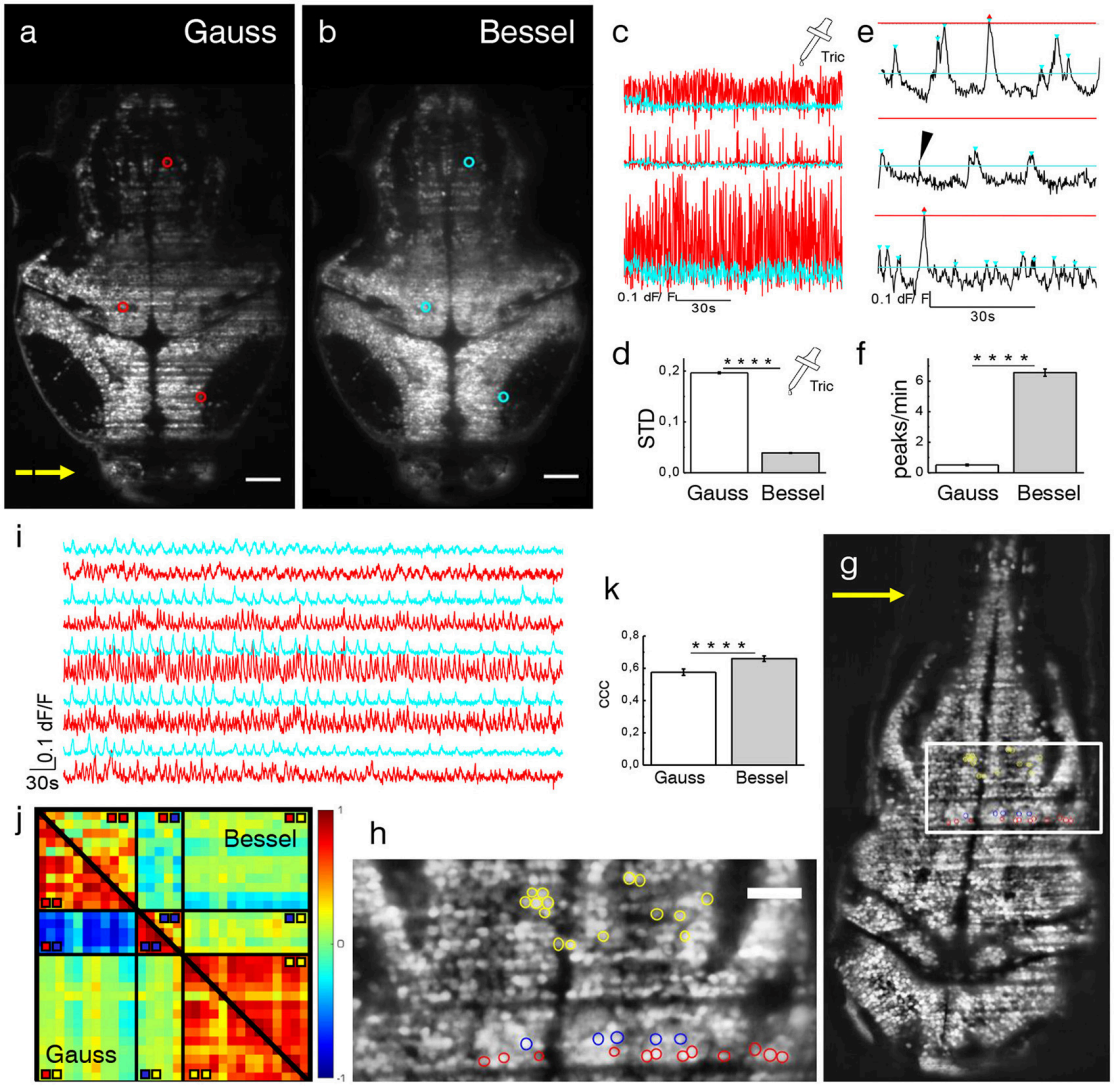
Ca2+-imaging. Transverse plane of a 4 dpf elavl3:H2B-GCaMP6s larva imaged with Gaussian **(a)** and Bessel beam illumination **(b)**. Scale bar is 50 μm. Yellow arrow indicates direction of light sheet. Indicated are three exemplary cells with red (cyan) circles which have been illuminated with a Gaussian (Bessel) beam and whose traces can be seen in **(c)**. **(d)** Mean standard deviation (STD) of traces without neuronal activity (*p* < 0.0001, paired *t*-test, *n* = 625 cells in *N* = 7, error is sem). **(e)** Exemplary dF/F traces measured with Bessel beam illumination during neuronal activity. Indicated are peaks (triangles) above the noise level for Gaussian (red) and Bessel beam illumination (cyan). Peaks with prominence below threshold were discarded (black arrow head). **(f)** Peaks per minute detected above noise level using appropriate thresholding to exclude Gaussian and Bessel baseline noise (*p* < 0.0001, paired *t*-test, *n* = 586, in *N* = 1 larva, error is sem). **(g)** Transverse plane of a 4dpf larva imaged with Gaussian illumination. Indicated are cells located on two adjacent excitation lines (red, blue) and randomly in the hindbrain as part of an active network (yellow, see also zoomed view in **h**). **(i)** Exemplary traces of cells (marked yellow in **h**) measured with Gaussian (red) and Bessel beam illumination (cyan). **(j)** Correlation matrix of cells measured with Gaussian (lower triangular) and Bessel beam illumination (upper triangular matrix). The color scale ranges from –1 (blue) to +1 (red). **(k)** Averaged coefficient of cross correlations (ccc) of yellow yellow quadrant (*p* < 0.0001, paired *t*-test, *n* = 91, *N* = 1, error is sem).

Due to the irregular flow of blood cells, the traversing excitation light sheet is either absorbed or scattered out of its trajectory such that the baseline fluorescence in adjacent neurons is no longer steadily generated, both because of direct shadowing of in-focus fluorophores and of spurious excitation of out-of-focus ones. In the following, dF/F traces of pairwise identical neurons, imaged with either a Gaussian or Bessel beam, located in areas affected by strong flickering and located as described in the previous section, were compared. Larvae were treated with tricaine (160 mg l-1), a commonly used general anesthetic to globally lower neuronal activity (Attili and Hughes, 2014; Turrini et al., 2017), to ensure that any changes in baseline fluorescence were only due to dynamic flickering and not neuronal activity. A transversal section of the encephalon of a 4 dpf Tg(elavl3:H2B-GCaMP6s) zebrafish larva is shown in Figures 4a,b. Three representative neurons are marked with red (cyan) circles for the Gaussian (Bessel) case and their dF/F traces are shown in Figure 4c. Different positions in the encephalon display different levels of baseline noise; however, in general, the noise obtained in Bessel traces was consistently lower than that in traces obtained with Gaussian illumination. Quantified by their standard deviation, we analyzed traces measured with Gaussian and Bessel beam illumination respectively to obtain the level of baseline noise in the absence of neuronal activity (Figure 4d). The baseline noise was 19.65 ± 0.18 % of dF/F for Gaussian and 3.90 ± 0.07 % for Bessel beam illumination (*p* < 0.0001, paired *t*-test, *n* = 625 cells, 15 time lapses at various depths in *N* = 7 larvae of 4–5 dpf, error is sem) constituting a 5-fold increase in sensitivity.

#### Statistical analysis of reduced sensitivity and accuracy

The previously reported increase in baseline noise directly reduces the sensitivity to calcium transients. In this section, using statistical arguments, we quantify the loss in peak detection and activity correlation due to time-varying hemodynamic flickering.

Exemplary traces in Figure 4e illustrate a peak counting routine (see Methods 15) which counted peaks above Gaussian baseline noise (red triangles) and again above Bessel beam baseline noise (cyan triangles) on identical traces obtained with Bessel beam illumination. Peaks of sub-threshold prominence (black arrow head) were discarded. The total number of peaks per minute detected above their respective baseline noise levels are reported in Figure 4f (*p* < 0.0001, paired *t*-test, *n* = 586 cells, in *N* = 1 larva, error is sem). The cells were located at 12 different depths throughout the encephalon of a 4 dpf larva. Whereas on average 6.6 peaks per minute could be detected above Bessel beam noise levels, only 0.5 peaks per minute were high enough to surpass the Gaussian baseline noise. This means that, statistically, if a given cell were to be located in an area affected by strong flickering, then less than 1 out of 12 peaks would be detected due to the higher associated baseline noise when using Gaussian compared to Bessel beam illumination.

To further illustrate the influence of increased baseline noise on dF/F traces, we generated random white noise of an amplitude corresponding to the Gaussian and Bessel baseline noise respectively, and multiplied it pairwise to traces obtained with Bessel beam illumination, see Figure S5. The native data set without synthetic noise was considered as ground truth and allowed an absolute comparison between Gaussian and Bessel beam illumination. The absolute cross correlation coefficients reported in Figure S5d reveal that while the mean of the Gaussian and native data set differ significantly (0.0978 vs. 0.1309), the difference between the mean of the Bessel and the native data set is not statistically significant (0.1308 vs. 0.1309) (*p* < 0.0001, *t*-test, *n* = 27,7885 corresponding to 746 cells in *N* = 1 larva, error is too small to be displayed). Next, the difference in cross correlation coefficients between the native traces and the traces with synthetic noises was calculated (Figures S5e,f,i). The histograms of these distributions give some important insights; whereas the mean value represents the bias of each modality, the standard deviation gives a measure of its accuracy. For Gaussian illumination, we obtained a mean value of 0.0127 (Figure S5g) whereas this value is 5.29E-5 for Bessel beam illumination (Figure S5j). This result indicates the introduction of a systematic error by Gaussian illumination which is approximately one tenth of the average absolute correlation coefficient. Furthermore, the standard deviations of the histograms give a direct measure of the random error introduced by each method and therefore a measure of accuracy. The standard deviation was 0.0682 for Gaussian and 0.0033 for Bessel beam illumination (Figure 4g, Figure S5e) which constitutes an 20-fold increase in accuracy compared to Gaussian illumination.

#### Real neuronal correlation is masked by spurious correlation

The previous section evaluated the reduction of sensitivity from a statistical point of view by distinguishing peaks with respect to numerical thresholds and by including baseline noise of typical amplitude both obtained by averaging hundreds of cells affected by flickering. We showed that the contamination with both systematic and random errors is significantly higher when using Gaussian beam compared to Bessel beams. In the following section, we present measurements of spontaneous neuronal activity in individual cells in the presence of flickering in one exemplary larva.

As indicated in Figures 4g,h, several cells have been manually selected in the hindbrain of a 4dpf larva to generate a scenario in which spurious correlations due to flickering significantly alter the correlation amongst cells in a circuit. The red and blue cells where chosen in an area determined by the methodology described in Figure 3e and corresponded to lines in which nearby flowing blood cells cast alternating shadows whereas the yellow cells have been randomly chosen among a circuit of cells that showed strong spontaneous activity (see Videos S5, S6). It is apparent from the correlation matrix (Figure 4j) that, when using Gaussian illumination, a strong correlation between cells on one line (sector marked 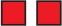 in the correlation matrix) can be observed which strongly anti-correlates with cells from the line immediately next to it (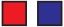), an obvious hemodynamic artifact. Notably when using Bessel beam illumination this strong spurious correlation is not observed (average cross correlation –0.571± 0.025 for Gauss vs. –0.013 ± 0.023 for Bessel beam illumination, *n* = 55, error is sem, see table in Figure S6). Exemplary traces of spontaneous activity outside an area strongly affected by flickering (cells marked yellow) are shown in Figure 4i. It is worthwhile pointing out, that when looking at correlations entirely outside flickering areas (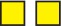), average correlation obtained with Gaussian beams was 0.576 ± 0.020 and 0.660 ± 0.017 (an increase of 15 %, *n* = 91) for Bessel beams (Figure 4k). This result demonstrates that even when steering clear of problematic areas with evident strong flickering, correlations of neuronal activity can be significantly affected by residual flickering, less evident to the human eye, when using Gaussian illumination. Since Bessel beams have a fivefold increased sensitivity to calcium transients compared to Gaussian beams, they are more likely to detect calcium transients in multiple functionally connected cells and this leads to a higher average correlation as seen in Figure 4j. A more comprehensive analysis based on automatic segmentation rather than manual selection of cells is presented in Figure S7.

### principle component analysis of spontaneous activity

**Figure 5:**
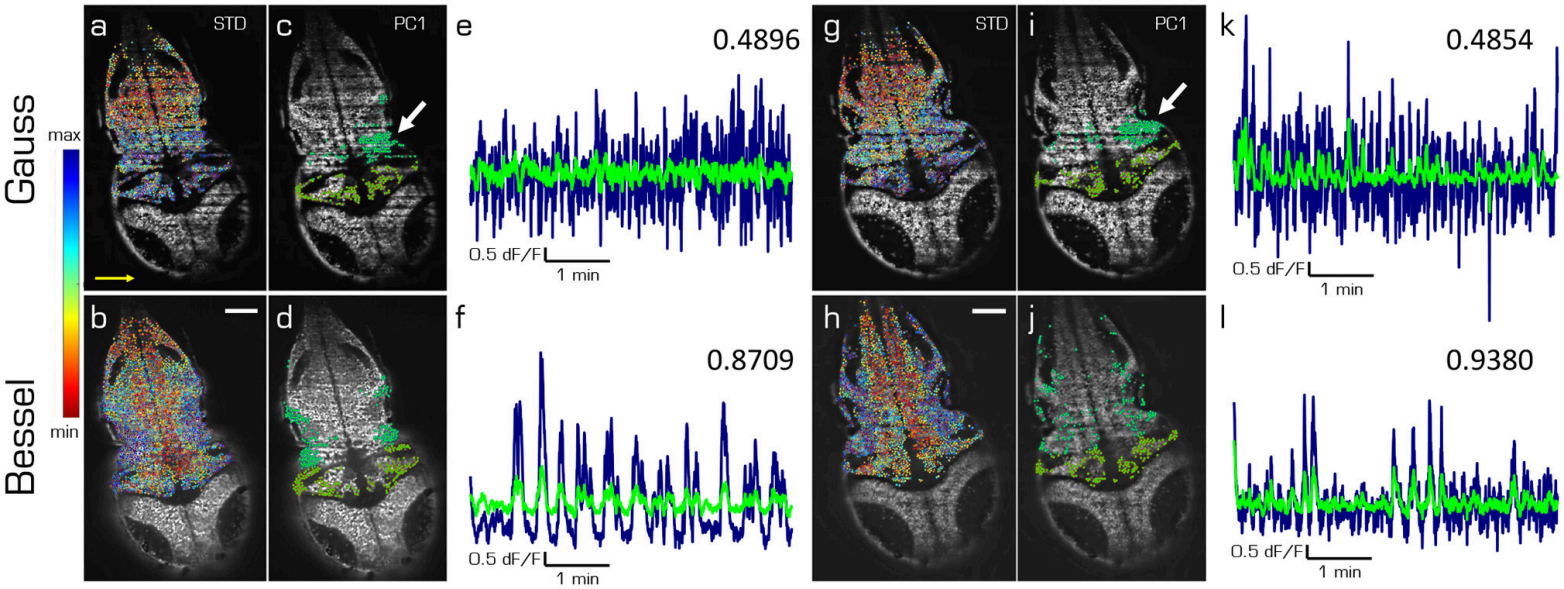
Principal components of spontaneous activity in the hindbrain and the cerebellum. Transverse planes of a 5dpf elavl3:H2B-GCaMP6s larva imaged with Gaussian (top) and Bessel illumination (bottom row) at two different depth (left and right half). Scale bar is 50 μm. Yellow arrow indicates direction of light sheet. **(a,b,g,h)** Standard deviation of the mean correlation to every other cell color-coded from blue (maximum) to red (minimum). **(c,d,i,j)** Indicate the 10 % of cells most contributing to the first principal component in the hindbrain and cerebellum respectively. **(e,f,k,l)** Time traces of the first principal component of the hindbrain (navy) and the cerebellum (light green) and the correlation between both traces (top right).

In this last section we compare the first principal components of spontaneous activity in the hindbrain and the cerebellum for Gaussian (top) and Bessel beam illumination (bottom row of Figure 5) at two different depths (left and right hand side) in one exemplary larva. The cells in Figures 5a,b,g,h are color-coded according to the standard deviation of their mean correlation with every other cell. While the emerging pattern shows lateral symmetry over the rostral-caudal axis of the larva for Bessel beam illumination, the pattern using Gaussian illumination is quasi identical in color along the illumination direction of the light sheet and mostly following a symmetry along the dextro-sinister axis of the larva. The 10 % of cells mostly contributing to the first principal component of the hindbrain and cerebellum respectively are shown in Figures 5c,d,i,j. Whereas with Bessel beam illumination a symmetric distribution of cells is apparent, Gaussian illumination leads to an agglomeration of cells mainly located on the far side with respect to the illumination and clustered around strong shadows which indicate the presence of flickering artifacts (white arrows). Accordingly, the time traces of the first principal component of the hindbrain (navy) and the cerebellum (green, Figures 5e,f,k,l) are less noisy and show higher correlation with each other when obtained from measurements with Bessel beam compared to Gaussian illumination.

## 3. Discussion

Large-scale neuronal recordings and their interpretation have been a paradigm shift toward understanding circuit function during behavior (Ahrens et al., 2012). With substantial improvements in imaging technology and a concurrent increase in the size and complexity of neuronal data, pressure now shifts toward data analysis as a fundamental bottleneck for neuroscience (Freeman et al., 2014). A common approach in large-scale imaging in zebrafish larvae is to apply an automatic segmentation algorithms which identifies ROIs associated with individual neurons (Panier et al., 2013; Kawashima et al., 2016) and extracts the dF/F traces from the contained pixel values.

Isolating fluorescent features of interest in a heterogeneous background places higher computational demands on the algorithms used, often with concurrent increase in computation time and complexity of the parameters to be tuned. A recent study showed that very simple algorithms like global thresholding or high pass filtering require uniform background intensity and fail to segment simple fluorescent forms like cell nuclei when a striated background simulating muscle fibers is added to the image (Chitalia et al., 2016). As the complexity of the fluorescent feature increases, so do the demands on the algorithms tasked to isolate them. Another recent study compared automated segmentation of a simple synthetic interrupted tube with progressively added salt and pepper noise by a range of published algorithm and their failure to accurately trace this simulated neurite at noise levels of five percent (Liu et al., 2016). In Figure 2, we show how a threshold of 5 % leads to more than a three quarters of the larva encephalon to be affected by a striated background, jeopardizing hours of microscope acquisition time, data post-processing and sample preparation if the data cannot be accurately segmented in an automated fashion.

Furthermore, the results presented in this paper demonstrate the contamination of data by artifacts created by hemodynamic flickering that threaten the automated extraction of calcium transients over the entire larva encephalon. Here, we have shown a reduction of the flickering area by a factor of ≈ 30 when using Bessel beam illumination (Figure 3). Whereas a previous publication (Panier et al., 2013) manually excluded severely affected neurons from further analysis, using Bessel beams would allow to include substantially more cells in a move from large-scale imaging toward true brain-wide analysis.

The deleterious effect of flickering artifacts caused by passing blood cells has been previously reported (Panier et al., 2013) but not quantified. Here, we present a statistical analysis to estimate the baseline noise associated hemodynamic modulation in most adversely affected neurons and report a STD of ≈ 20 % dF/F for Gaussian illumination compared to ≈ 4 % for Bessel beams. This represents a 5-fold increase in sensitivity to accurately reveal Ca2+ transients when using Bessel beam illumination that otherwise would have been buried in noise.

Notably, this sensitivity gain cannot be achieved simply by using standard double-sided illumination (Ahrens et al., 2012). Indeed, in the hypothesis of Gaussian noise distribution, the process of summing up two independent variables (the two illuminations) increases the signal-to-noise ratio only by a factor $\sqrt{2}$, about 3.5 times less than what we found with Bessel beams. The use of two-photon excitation in the light-sheet microscope (Wolf et al., 2015; Piksarv et al., 2017) might mitigate the problem, but is prone to shadowing artifacts even more than Bessel beam illumination (Fahrbach et al., 2013b). Furthermore, upgrading a single-photon LSM to Bessel beam illumination is substantially less expensive than introducing two-photon excitation.

The STD values presented here represent a worst-case scenario; in the best case, at least in 2D studies, the plane of interest contains no or very little vessels and therefore no flickering and consequently the baseline noise in Gaussian and Bessel beam are identical. For true 3D brain-wide acquisitions however, it will be impossible to fully avoid hemodynamic contamination.

To illustrate the effect of increased baseline noise, we have presented a statistical analysis applying peak counting and cross correlation to identical traces. By counting peaks due to spontaneous neuronal activity above the Gaussian baseline noise and the Bessel baseline noise we estimated that less than 1 out of 12 peaks would be detected. This is a conservative estimate since usually higher thresholds of signal to noise ratio (SNR), e.g., Rose criterion: SNR=5 (Rose, 1973), are common. Furthermore, using synthetic white noise, we have demonstrated that the randomizing effect of hemodynamic noise leads to a loss of correlation which cannot be hoped to be recovered by clever engineering of ever brighter calcium indicators due to its multiplicative nature.

Blood flow in the brain is regulated to match neuronal demand based on activity by restricting and dilating the diameter of vessels, a phenomenon known as neurovascular coupling (Attwell et al., 2010). Assuming HagenPoiseuille law of fluidic dynamics, this results in varying volumetric flow rates and therefore speeds of red blood cells. This plus other randomizing effects like vessel orientation and location means that neurons more than just a few cell diameters away from each other experience entirely uncorrelated noise. More strikingly however, the opposite is true as well: neurons which lie in close proximity on a line parallel to the excitation light will experience a baseline noise that is very strongly correlated and capable of generating spurious correlations that are not due to activity. Playing the devil's advocate, we “created” a correlation matrix in which the positive correlation between cells on one line and their anti-correlation with cells on a neighboring line were so strong as to mask actual correlation due to activity in an area not severely affected by flickering. This result is important because it shows that not only do Bessel beams reduce the area affected by strong flickering in which they also generate a significantly lower baseline noise. Additionally, even in areas that are not strongly affected by flickering due to immediately close by vessels, Bessel beams reveal neuronal activity that is lost when using Gaussian illumination.

We point out that while PCA is not suited to isolate hemodynamic flickering due to the dependent nature of the components, independent component analysis (ICA) has been used previously to identify various artifact sources (Vigário et al., 1998) and might be useful not only to contain flickering artifacts but also to extract information on local blood flow.

A prerequisite to understanding how the brain interacts with the outside world is to understand its intrinsic spatio-temporal dynamics in complete absence of external stimuli. Activity patterns are spontaneously produced in the brain and have been demonstrated to influence brain function (Romano et al., 2015), which is why neuronal interactions underlying spontaneous activity and its biological relevance have been gaining interest in recent years (Ringach, 2009; Deco et al., 2011, 2013; Destexhe, 2011).

This complete absence of external stimuli has a far-reaching consequence for the imaging setups designed to reveal the underlying mechanism of spontaneous activity. It is fundamentally impossible to average responses over triggering trials to clean up the signal. Single one-shot measurements of functional traces therefore need to be intrinsically clean enough to allow extraction of meaningful data without averaging. In Figure 5 we have demonstrated how hemodynamic noise leads to a reduction to 56 % of correlation between the first principal component of the hindbrain and the cerebellum when using Gaussian compared to Bessel beam illumination.

Although we do not claim biological reproducibility as only one fish has been investigated, these results serve as a proof of principle that applying Bessel beams to quantitative light-sheet microscopy is indeed an enabling technique capable to reveal correlations in functional traces that might otherwise remain buried in noise.

In conclusion, we have illustrated how flickering can adversely affect and even falsify data extracted from LSM images. We compared the performance of Gaussian and Bessel beam illumination in functional studies employing Ca2+ imaging in larval zebrafish. We have identified sources of contamination in the form of true correlation lost in a multiplicative baseline noise and spurious correlation overpowering correlation due to actual neuronal activity when using standard illumination. We have shown how the use of Bessel beams can provide an optical solution to correct for these artifacts on the microscope system side and allow for high-fidelity imaging in light-sheet microscopes. The results given here present a new quality standard for quantitative single-neuron sensitivity measurements in LSM opening up novel experiments on spontaneous activity.

## 4. Materials and methods

### Zebrafish husbandry and larva mounting

We generated two stable zebrafish transgenic lines using a Tol2 construct with elavl3 promoter that drives the expression of the genetically encoded calcium indicator GCaMP6s in all neurons (Freeman et al., 2014). Tg(elavl3:GCaMP6s) line showed a cytoplasmic expression of the transgene whereas line Tg(elavl3:H2B-GCaMP6s) expressed GCaMP6s in fusion with histone H2B and consequently showed nuclear localization. Larvae were kept according to standard procedures (Westerfield, 1995). Each 4-5dpf Tg(elavl3:H2B-GCaMP6s) larva, of unknown sex, used in the experiments was transferred into a reaction tube containing 1.5 % low gelling temperature agarose (A9414, Sigma) in fish water (150 mg Instant Ocean, 6.9 mg NaH 2PO 4, 12.5 mg Na 2HPO 4 per 1 l of dH 2O), kept at 38 °C. The zebrafish larva was then drawn with a syringe into a glass capillary (O.D. 1.5 mm); after gel polymerization, the agarose cylinder containing the larva was gently extruded until the head protruded from the glass (Figure S1b). The glass capillary was then mounted onto an x−, y−, z−, Θ-stage (M-122.2DD and M-116.DG, Physik Instrumente, Germany) and immediately immersed into the sample chamber containing fish water. The fish water was kept at a constant temperature of 28.5 °C.

### Calcium imaging

For *in-vivo* Ca2+ imaging a line exposure time of 0.3 ms was used, resulting in a frame rate of 44 Hz. When using Bessel beam illumination, power was increased by a factor of three compared to Gaussian illumination. Typical laser power at the back focal plane of the excitation objective was 0.7 mW for Gaussian illumination and 2 mW for Bessel illumination. These power levels have been already reported in the literature to be safe in term of photo-toxicity (Panier et al., 2013). The 16bit depths images constituting each time lapse were first converted from the proprietary Hamamatsu file format to a multi-page tiff and simultaneously temporally down sampled by a factor of 8 to save disc space resulting in an effective frame rate of 5.5 Hz. When tricaine was added to the fish water and the traces therefore were used to analyze flickering, no temporal downsampling was performed. Z profiles of regions of interests were then extracted with a FIJI macro over a hand selected circular area covering the cell nucleus or else using a Matlab script for automated segmentation (Kawashima et al., 2016). The dF/F traces were obtained with a custom-written Matlab script in which, firstly, the background, measured over a ROI outside the larva on the proximal side to the laser, was subtracted and secondly, the baseline of the traces were calculated using the *msbackadj* function. Thirdly, the baseline was subtracted from the trace and finally, the trace was divided by the baseline to normalize it (Freeman et al., 2014).

### Estimation of the area affected by flickering

The area affected by primary flickering for Gaussian and Bessel beam illumination was estimated in a plane by using a sequence of custom-made macros (FIJI) and programs (LabVIEW). By primary flickering is intended strong variations in baseline fluorescence intensity directly attributable to a large, nearby blood vessel. The more subtle, yet noticeable, effect of smaller peripheral blood vessels was neglected as it was much harder to quantify. Using this automated method 2D maps of severe flickering areas were obtained which allowed us to calculate the percentage of the entire encephalon that were prone to a substantial source of additive noise. To assure that the evidenced changes in pixel brightness were not due to activity, Tricaine (160 mg l-1), a general anesthetic that blocks voltage sensitive Na+ channels preferentially in neurons, was added to the fish water.

The methodology (Figure S3), based predominantly on a temporally down-sampled time lapse in which each frame corresponded to the standard deviation (STD) of a certain frame window of the original raw data, evidenced the characteristic shape of long horizontal stripes. A custom-written FIJI macro was used to calculate the STD time lapse of the raw data time lapse with window size 5 and step size 5. After adjusting both time lapses to the same brightness scale, an additional macro automatically compared the STD time lapses of the Gaussian and Bessel beam by running through a sequence of functions to enhance the features of the images that changed most in brightness value using the exact same parameters in both cases. With both time lapses displayed on the same brightness scale, first a gamma of 1.1, second a bandpass filter and third a variance filter were applied over the entire image. After again adjusting both time lapses to the same brightness scale, each time lapse was collapsed to a single image using using a z projection of the STD. The resulting images were thresholded to a common value and converted to 8 bit binary where dark spots indicating the parts of the encephalon that had changed the most during high frame rate (44 Hz) acquisition. The binary images where then analyzed in a custom-written LabVIEW program that automatically found the bounding boxes of each identified particle (Figure S4). In the case of Gaussian illumination, the horizontal dimension of the bounding box of each particle was extended up to a mask corresponding to the free-hand selection of the encephalon of the larva. In the case of the Bessel beam illumination, the bounding box was extended to a length corresponding to the theoretically calculated reconstruction length of the Bessel beam which follows from simple trigonometric considerations:

(1) $$\begin{array}{l} x=\frac{\tan\beta }{0.5*h} \end{array}$$

where β is the angle of the cone in the sample chamber, and *h* is the height of the bounding box. The LabVIEW program then automatically calculated the sum of all bounding boxes and their percentile of the free-hand selection of the entire encephalon. The characteristic horizontal stripes of the resulting areas, confirmed that the method indicated primarily shadowing artifacts and not individual neuronal activity.

### Peak counting and correlation analysis

For peak counting, the dF/F traces were loaded into a custom-written Matlab program and smoothed using the *sgolayfilt* function (order 5, framelen 7). The *findpeaks* function was then used to automatically detect peaks above the Gaussian and Bessel noise level respectively with a minimum peak prominence of 8.5 % of dF/F. The correlation matrices were calculated using a custom-written Matlab program using the *corr2* function. Noise (see Figure S5) was generated according to the following pseudo Matlab code to produce random noise that oscillates around an average of unity:

*noise* = STD .* randn + 1;

The noise vector was than point-wise multiplied to the trace vector which was averaged to zero using:

*y* = *(trace* - μ) .* noise;

### Principle component analysis

For principle component analysis, all time lapses were temporally downsampled by a factor of 10 to a final frame rate of 4.4 Hz, binned (2 × 2, average) and converted to 8bit. The resulting time lapses were registered to a reference frame using the TurboReg plugin in FIJI. After automated segmentation using the script in Kawashima et al. (2016) and extraction of the dF/F traces for all segmented cells, the principal components were calculated using the *pca* function in Matlab.

### Experimental design and statistical analysis

To assess the area affected by flickering, which we assumed would have some variability between larvae, a sample size of N=10 larva was chosen. To assess the baseline contamination with random noise, again assuming some variability between larva, but mostly expecting variability between different cells, a sample size of N = 7 larva was used. To obtain “native” traces of spontaneous activity in which we counted peaks above certain thresholds and to which we added synthetic noise, we concentrated our study on the variability between cells taking advantage of the single cell resolution afforded by the microscope, which is why a sample size of N = 1 larva was used. The cross correlation of traces of spontaneous activity in areas affected by strong flickering represents a specifically constructed case study, which is why a sample size of N = 1 larva was used. The comparison between the principle components of spontaneous activity were exemplified in N = 1 larva specifically to demonstrate the strength of Bessel beam illumination for one-shot experiments. Each experiment was performed first in Gaussian modality and then immediately again in Bessel beam modality. An experiment constitutes the acquisition of a time-lapse. Experiments were repeated on several transversal planes in the larva encephalon. The biological replication of experiments occurs each time we changed the larva. The technical replication occurs when experiments were repeated in the same larva but in a different transversal plane. There were no outliers. There was no inclusion/exclusion of data. Each graph clearly states the statistical analysis method used (mostly paired t-test to directly compare Gaussian and Bessel illumination), the sample size N (animals) or n (cells/planes), the mean, the standard error of the mean and the *p*-value in the figure legend as well as in the main result text.

## Ethics statement

All experiments were carried in accordance to Italian law on animal experimentation (D.L. 4 March 2014, n.26), under authorization n. 407/2015-PR from the Italian Ministry of Health.

## Author contributions

MM, LSi, and LSa conceived the experiments. LT, FV, and NT generated the transgenic zebrafish lines. LT prepared the samples. MM, TA, and AG built the microscope. MM and LT conducted the experiments. MM analyzed the results. MM wrote the manuscript with input from all co-authors. FP acquired all funding and supervised the project.

### Conflict of interest statement

The authors declare that the research was conducted in the absence of any commercial or financial relationships that could be construed as a potential conflict of interest.
