# Corrigendum: Bessel Beam Illumination Reduces Random and Systematic Errors in Quantitative Functional Studies Using Light-Sheet Microscopy

**DOI:** 10.3389/fncel.2019.00025

**Published:** 2019-02-22

**Authors:** M. Caroline Müllenbroich, Lapo Turrini, Ludovico Silvestri, Tommaso Alterini, Ali Gheisari, Natascia Tiso, Francesco Vanzi, Leonardo Sacconi, Francesco S. Pavone

**Affiliations:** ^1^National Institute of Optics, National Research Council, Sesto Fiorentino, Italy; ^2^European Laboratory for Non-linear Spectroscopy, LENS, Sesto Fiorentino, Italy; ^3^Department of Biology, University of Padova, Padua, Italy; ^4^Department of Biology, University of Florence, Sesto Fiorentino, Italy; ^5^Department of Physics and Astronomy, University of Florence, Sesto Fiorentino, Italy

**Keywords:** spontaneous activity, zebrafish, principle component analysis, light-sheet microscopy, functional imaging, Bessel beams, flickering artifacts, striping

Natascia Tiso was not included as an author in the published article. The corrected Author Contributions Statement appears below.

“MM, LSi, and LSa conceived the experiments. LT, FV, and NT generated the transgenic zebrafish lines. LT prepared the samples. MM, TA, and AG built the microscope. MM and LT conducted the experiments. MM analyzed the results. MM wrote the manuscript with input from all co-authors. FP acquired all funding and supervised the project.”

Additionally, the “Department of Biology, University of Padova, Padua, Italy” has been added as Natascia Tiso's affiliation in the published article.

Lastly, the corresponding email address for the author “M. Caroline Müllenbroich” has been changed to “caroline.muellenbroich@glasgow.ac.uk.”

The authors apologize for these errors and state that this does not change the scientific conclusions of the article in any way. The original article has been updated.

