# Supplementary material for: Bessel Beam Illumination Reduces Random and Systematic Errors in Quantitative Functional Studies Using Light-Sheet Microscopy
Source: Front Cell Neurosci. 2018 Sep 20;12:315. doi: 10.3389/fncel.2018.00315 (PMC6158350; doi:10.3389/fncel.2018.00315)
Supplement: Supplementary file 7 [file Image_1.PDF]

Supplementary Material: Bessel beam illumination reduces random and systematic errors in quantitative functional studies using light-sheet microscopy

BESSEL BEAMS

The paraxial Helmholtz equation governs diffraction phenomena and, most typically, its solution describes the propagation of electromagnetic waves in the form of Gaussian beams characterized by a transverse intensity profiles following a Gaussian distribution. Alternative solutions to the Helmholtz equation exist in the form of diffraction-free modes, most notably Bessel beams (Durnin et al. (1987)), whose central core can be extremely narrow without being subject to diffraction (McGloin and Dholakia (2005)). Due to finite apertures necessarily limiting the realization of such beams experimentally, only Quasi-Bessel beams can be generated for which the propagation invariance holds true over a finite propagation range (Durnin et al. (1988)). A simple way to create a Bessel beam is by superimposing plane waves whose wave vectors lie on a cone using a conical lens (Indebetouw (1989), Figure S1-a).

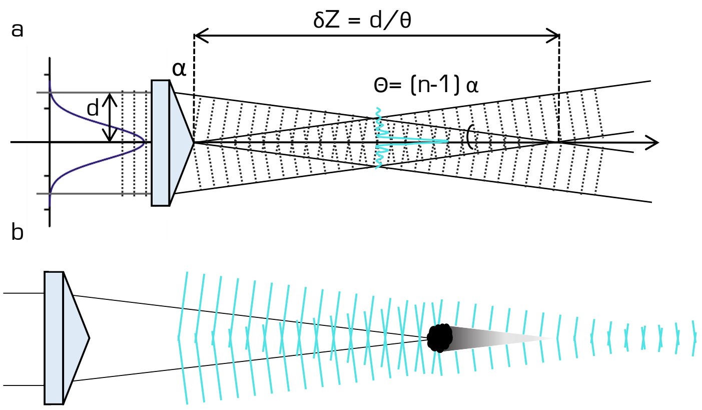

Figure S1. a) Generation of a Bessel beam using an axicon lens. The characteristic transverse J_0 Bessel beam profile of a central lobe with concentric rings is created within the propagation length δZ of the axicon. **b)** If an obstacle obstructs the central lobe of the Bessel beam, the optical power stored in the concentric rings can regenerate the initial beam profile in the reconstruction region behind the shadow zone.

The axicon creates the characteristic circularly symmetric beam profile of a tight central core with concentric rings within its depth of focus δZ given by:

$$\delta Z = \frac{d}{\Theta} \tag{S1}$$

where d is the radius of the Gaussian beam impinging on the axicon and Θ is the Bessel beam cone aperture depending on the refractive index n and angle of the conical lens α:

$$\Theta = (n-1)\alpha \tag{S2}$$

The radius of the central core is given by:

$$r_c = \frac{2.405\lambda}{2\pi \sin\theta} \tag{S3}$$

where λ is the wavelength of the excitation light.

Each concentric ring carries approximately the same energy as the central core (Durnin et al. (1988)) and it is the optical energy stored in the concentric rings that is capable of regenerating the beam profile if the central lobe is obstructed (MacDonald et al. (1996); Bouchal et al. (1998); Garces-Chavez et al. (2002), Figure S1-b) making Bessel beams robust to imaging in scattering media.

Axicons are cheap and readily available and additionally are polarization invariant, however, the round tip of the axicon produces a Gaussian contamination to the Bessel beam since it ultimately acts like a lens. The Gaussian contribution can be easily eliminated in a Fourier plane where the Bessel beam takes the form of a ring by using a circular mask (Brzobohatý et al. (2008)). Alternatively, Bessel beams can also be generated with spatial light modulators, be it at an increased cost.

The axial resolution of a LSM is dominated by the thickness of the light sheet, which, for high NA detection objectives, is usually larger than the depth of field of the detection objective. For Gaussian beam illumination, the thickness of the light sheet can be calculated according to formulas of Gaussian optics (Saleh et al. (1991)):

$$\omega_0 = \frac{\lambda f^{eff}}{\pi\omega} \tag{S4}$$

where ω is the $1/e^2$ radius of the beam entering the illumination objective, f^{eff} is the effective focal length of the illumination optics, and λ is the wavelength in the medium. Due to the limited depth of focus of a Gaussian beam, the thickness of the light sheet is not constant throughout the field of view but follows a hyperbolic function. The confocal parameter b, being the distance along which the beam radius remains smaller than $\sqrt{2}\omega_0$ and given by:

$$b = \frac{2\pi\omega_0^2}{\lambda}, \tag{S5}$$

is often matched to the field of view (FOV) of the LSM and therefore gives a limit to the axial resolution at a given FOV.

For Bessel beam illumination the FOV is given by the depth of focus δZ in eq. S1 while the axial resolution is somewhat harder to define as it is given not only by the thickness of the central core $2r_c$ (eq.S3) but also by the increasingly diminishing contribution of the outer rings (see projection of a Bessel beam in Fig. S2-h). While it is the optical energy stored in the outer rings gives rise to the self-healing capabilities of Bessel beams, it is also the cause of some disadvantages. The on-axis irradiance in Bessel beams is lower compared to peak Gaussian beams of the same power. Additionally the outer rings can generate out-of-focus fluorescence which means that, in general, images acquired with a Bessel beam exhibit lower contrast and inferior optical sectioning compared to Gaussian illumination. This out-of-focus contribution can be partially excluded by confocal line detection (Baumgart and Kubitscheck (2012); Fahrbach and Rohrbach (2012)) or avoided in combination with sectioned Bessel beams (Fahrbach et al. (2013)).

REFERENCES

Baumgart, E. and Kubitscheck, U. (2012). Scanned light sheet microscopy with confocal slit detection. *Optics express* 20, 21805–21814

Bouchal, Z., Wagner, J., and Chlup, M. (1998). Self-reconstruction of a distorted nondiffracting beam. *Optics Communications* 151, 207–211

Brzobohatỳ, O., Čižmár, T., and Zemánek, P. (2008). High quality quasi-bessel beam generated by round-tip axicon. *Optics Express* 16, 12688–12700

Durnin, J., Eberly, J., and Miceli, J. (1988). Comparison of bessel and gaussian beams. *Optics letters* 13, 79–80

Durnin, J., Miceli Jr, J., and Eberly, J. (1987). Diffraction-free beams. *Physical review letters* 58, 1499

Fahrbach, F. O., Gurchenkov, V., Alessandri, K., Nassoy, P., and Rohrbach, A. (2013). Self-reconstructing sectioned bessel beams offer submicron optical sectioning for large fields of view in light-sheet microscopy. *Optics express* 21, 11425–11440

Fahrbach, F. O. and Rohrbach, A. (2012). Propagation stability of self-reconstructing bessel beams enables contrast-enhanced imaging in thick media. *Nature communications* 3, 632

Garces-Chavez, V., McGloin, D., Melville, H., Sibbett, W., and Dholakia, K. (2002). Simultaneous micromanipulation in multiple planes using a self-reconstructing light beam. *Nature* 419, 145–147

Indebetouw, G. (1989). Nondiffracting optical fields: some remarks on their analysis and synthesis. *JOSA A* 6, 150–152

Kawashima, T., Zwart, M. F., Yang, C.-T., Mensh, B. D., and Ahrens, M. B. (2016). The serotonergic system tracks the outcomes of actions to mediate short-term motor learning. *Cell* 167, 933–946

MacDonald, R., Boothroyd, S., Okamoto, T., Chrostowski, J., and Syrett, B. (1996). Interboard optical data distribution by bessel beam shadowing. *Optics Communications* 122, 169–177

McGloin, D. and Dholakia, K. (2005). Bessel beams: diffraction in a new light. *Contemporary Physics* 46, 15–28

Saleh, B. E., Teich, M. C., and Saleh, B. E. (1991). *Fundamentals of photonics*, vol. 22 (Wiley New York)

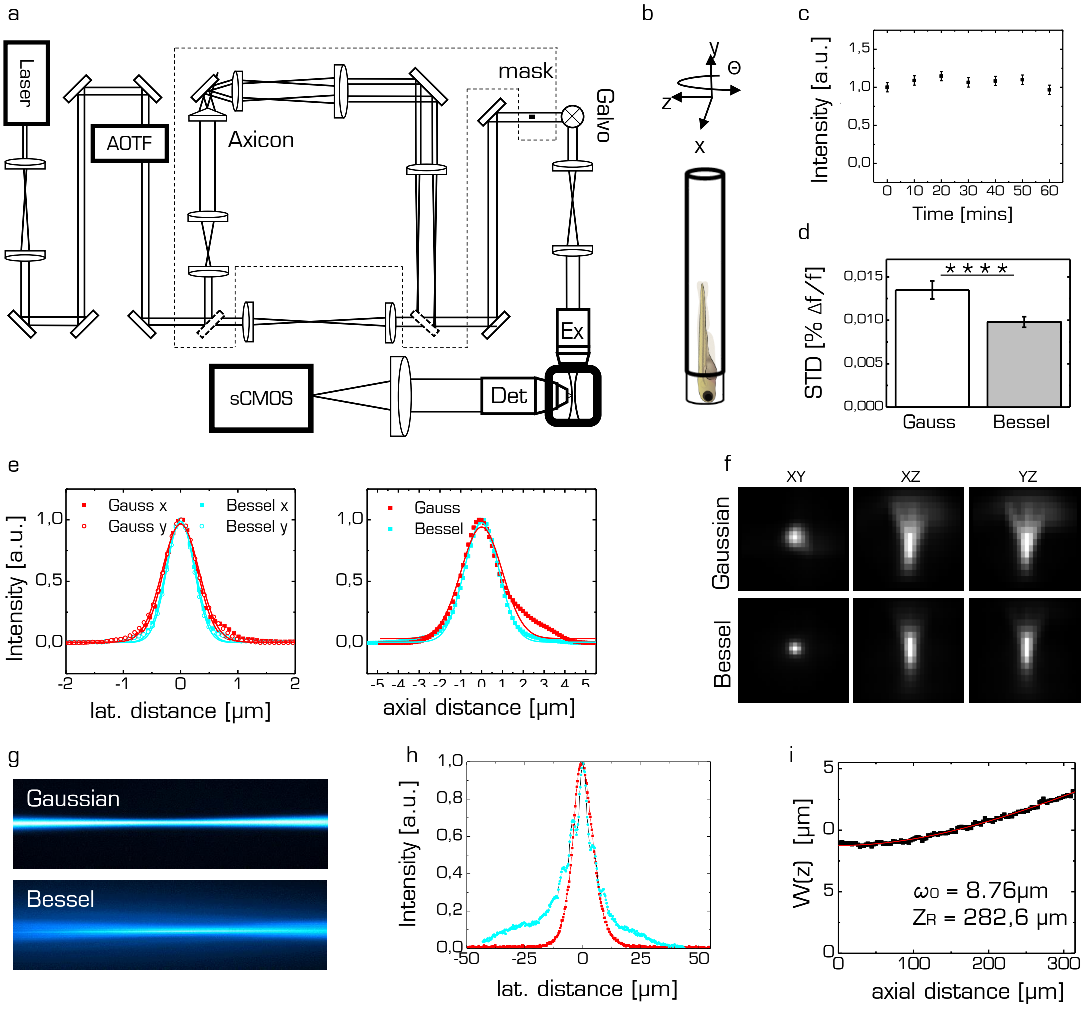

Parameter	Gauss	Bessel
Lateral FWHM of PSF (μm)	0.65<br>0.69	0.58<br>0.55
Axial FWHM of PSF (μm)	2.07	1.87
Confocal parameter (μm)	565	-
Self reconstructing length (mm)	-	1.5*
Beam NA (rad)	0.031	0.12*
Light-sheet thickness FWHM (μm)	10.3	-
Central lobe FWHM (μm)	-	2.64

Figure S2. a) Light-sheet microscope for functional imaging. **b)** Sample mounting: zebrafish larvae were embedded in low-melting point agarose and drawn with a syringe into a glass capillary. The capillary was mounted onto an x-, y-, z-, Θ-stage. **c)** Average image intensity during one hour exposure by the Bessel beam, error is standard deviation. No photobleaching is observed. **d)**. Standard deviation of the flickering measured with fluorescein in the sample chamber and by selecting ROIs the typical size of cells evenly distributed over the field of view. **e)** Measured point spread functions (PSFs) for the lateral and axial directions for Gaussian (red) and Bessel illumination (cyan) using fluorescent beads of $100\,\mathrm{nm}$ diameter. Beads were automatically extracted and distilled into a PSF using commercial software (PSF distiller, Huygens Software, Scientific Volume Imaging BV, Hilversum, The Netherlands). The full width half maximum (FWHM) of the PSF are reported in the table for Gaussian and Bessel illumination respectively. **f)** PSFs for Gaussian and Bessel illumination. **g)** Longitudinal beam profile. **h)** Transversal profile for the Gaussian (red) and Bessel beam (cyan). **i)** Beam width $\omega(z)$ of the Gaussian beam extracted from profile shown in (g). Red line indicates fit to hyperbolic function. The beam waist ω_0 and the Rayleigh range Z_R were extracted from the fit. Bottom: table of all beam parameters. * indicates theoretically derived values.

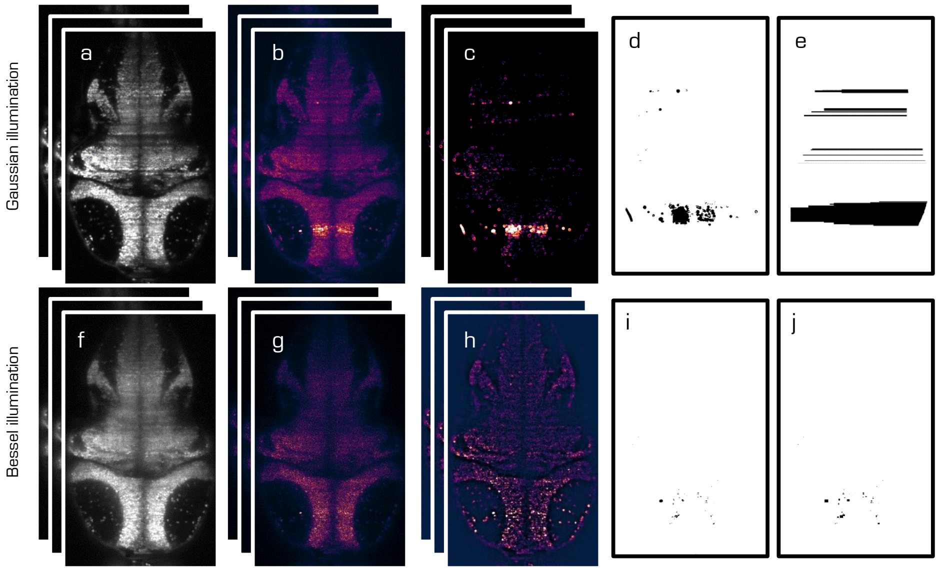

Raw data	Evidencing	Enhancing	Segmentation	Localization
Custom LabView	FIJI (ImageJ)			LabVIEW
Time lapse (16bit, 48Hz)	Time lapse (32bit, ca 10Hz) (5x temporal downsampling)		Single binary image (8bit)	
	Projection of STD windowsize: 5, stepsize: 5	Gamma, BPF, Variance filter	Z projection of STD, Thresholding	Estimation with bounding boxes

Figure S3. Work flow to estimate area severely affected by primary flickering. The first row refers to data obtained with Gaussian illumination while the second row was obtained with Bessel illumination. The raw data stack **(a,f)** was first temporally downsampled by calculating projections of the STD over 5 frame windows. Those STD stacks **(b,g)** were then treated over the entire image to further enhance the flickering areas, evidenced as bright horizontal stripes, using Gamma, Band path filter (BPF) and variance filter. The resulting time lapses **(c,h)** are projected along z to create single STD images, which were adjusted to the same brightness scale before a common threshold was applied to binarize them **(d,i)**. The binary images were then evaluated with a custom-written LabVIEW program to automatically localize the "blobs". The bounding boxes **(e,j)** were used to calculate the overall area affected by flickering. See methods for details.
(a): movie S8, raw data time lapse imaged with a Gaussian beam.
(f): movie S9), raw data time lapse imaged with a Bessel beam.
(b): movie S10, standard deviation time lapse imaged with a Gaussian beam.
(g): movie S11, standard deviation time lapse imaged with a Bessel beam.

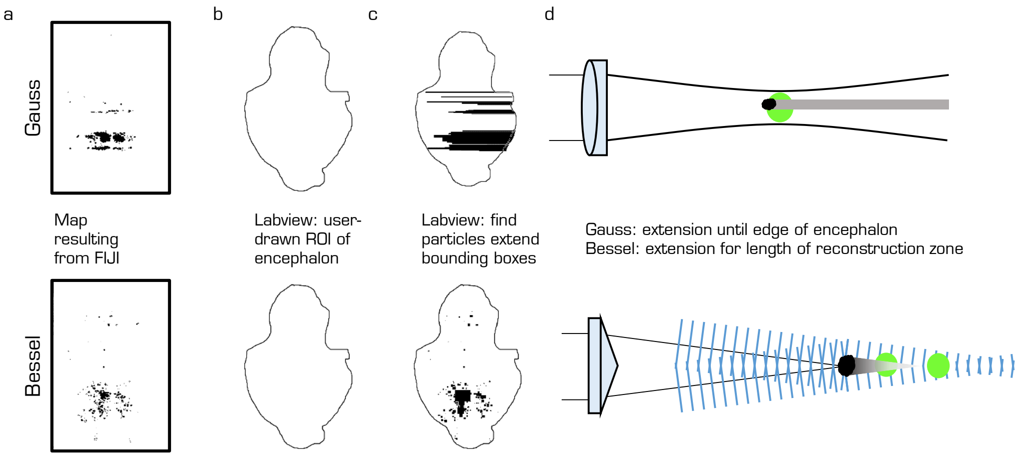

Figure S4. Extension of bounding boxes. The upper row refers to Gaussian illumination whereas the lower row refers to Bessel beam illumination. The binary image **(a)** obtained from the methodology illustrated in Fig.S3 was loaded into a custom-written LabVIEW program that automatically determined the bounding boxes of each identified particle. The only input of the user was a free-hand selection of the larva encephalon **(b)** that was used for both Bessel and Gauss evaluation. For Gaussian illumination the bounding boxes were horizontally extruded until they reached the limits of the encephalon mask **(c)** whereas for Bessel illumination the bounding box were extended up to a length corresponding to the reconstruction length of the Bessel beam in the sample plane. The LabVIEW program then automatically summed the area of all bounding boxes and their percentage of the entire encephalon. **(d)** Schematic on the casting of shadows for Gaussian and Bessel beam illumination.

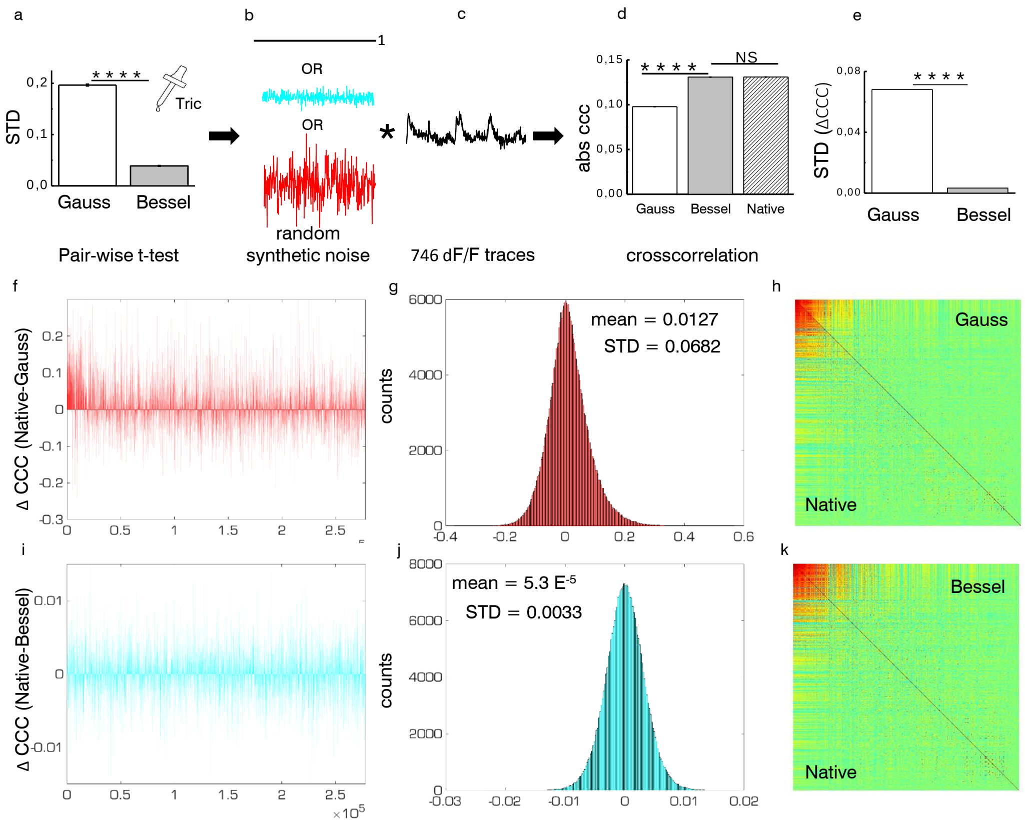

Figure S5. Correlation matrix showing the effect of random synthetic noise. The conceptual flow is as follows: from n=625 cells with inhibited activity (Tricaine) an average baseline noise was calculated using a pair-wise t-test for Gaussian and Bessel beam illumination respectively **(a)**. This noise level was used as amplitude to generate random white noise **(b)** which was point-wise multiplied to 746 dF/F traces measured with Bessel beam modality **(c)**. By defining the 746 synthetic noise-free traces as ground truth (native), we obtained three data sets that we compared by respective cross correlation. **(d)** Average absolute cross correlation coefficient (ccc) ($p < 0.0001$, t-test, n= 277885 corresponding to 746 cells, N=1, error is sem, NS: not significant).**(e)** STD of the difference in cross correlation coefficients between native traces and the same traces with multiplied noise. ($p < 0.0001$, paired t-test, n= 277885 corresponding to 746 cells, N=1). To obtain a measure of accuracy, we subtracted the Gaussian cross correlation coefficients from the native counterparts (**f**). The histogram of this distribution **(g)** yielded a mean indicating a bias of a measurement made in Gaussian modality, whereas the STD is a measure for its accuracy. **(h)** Correlation matrix showing the native traces (without synthetic noise) in the lower triangular matrix and the traces with multiplied Gaussian noise in the upper triangular matrix. **(i,j,k)**) Same as f,g,h) but for Bessel beam modality.

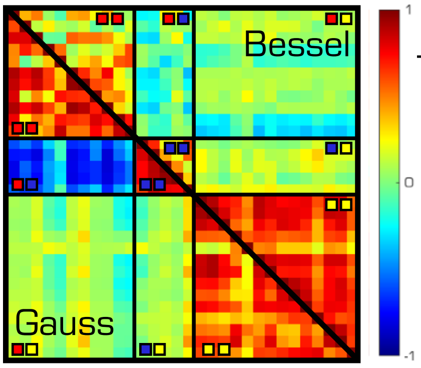

Corr	Gaussian	Bessel	p-value	n
■■	0.613 ± 0.032	0.292 ± 0.030	<0.0001	55
■■	-0.571 ± 0.025	-0.093 ± 0.023	<0.0001	55
■■	0.051 ± 0.010	-0.009 ± 0.012	<0.0001	154
■■	0.807 ± 0.030	0.391 ± 0.058	<0.0001	10
■■	0.111 ± 0.014	0.126 ± 0.011	0.2231	70
■■	0.576 ± 0.020	0.660 ± 0.017	<0.0001	91

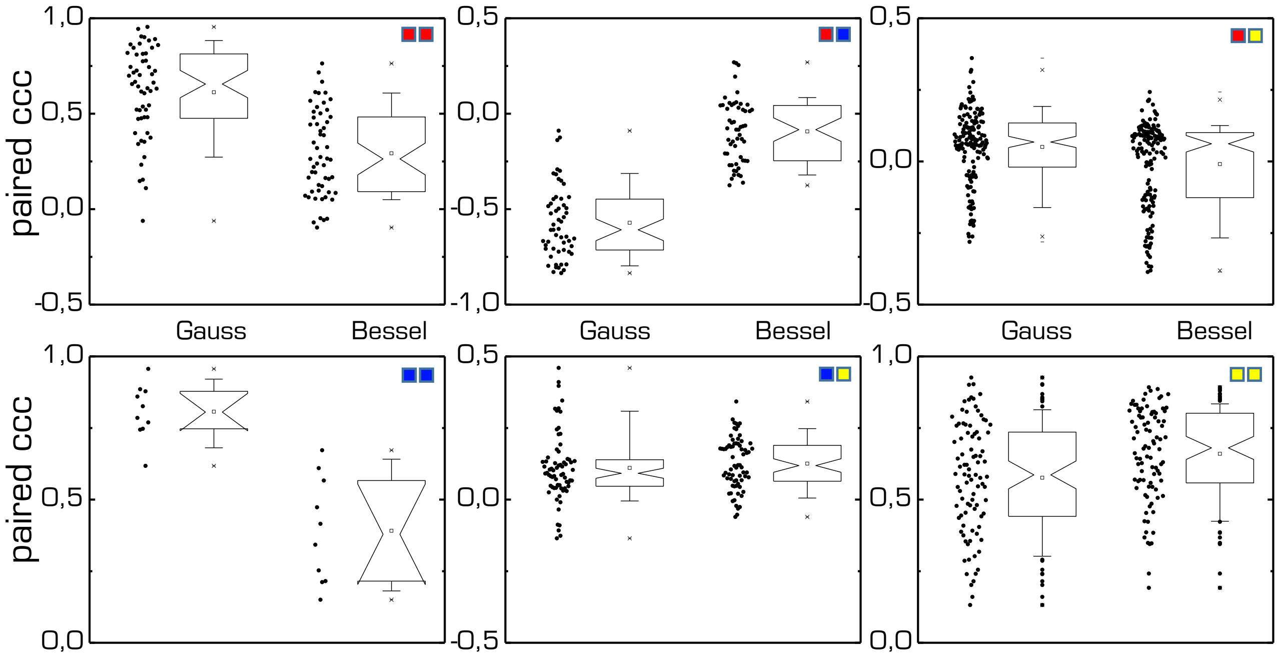

Figure S6. Comparison of the averaged cross correlation coefficients for groups of cells for Gaussian and Bessel beam illumination. Colors correspond to groups of cells marked in Fig.2-i). Table lists mean of paired t-test, error is sem. Box plots show corresponding data points.
see movie S12, raw data time lapse imaged with a Gaussian beam.
see movie S13), digital zoom into ROI.

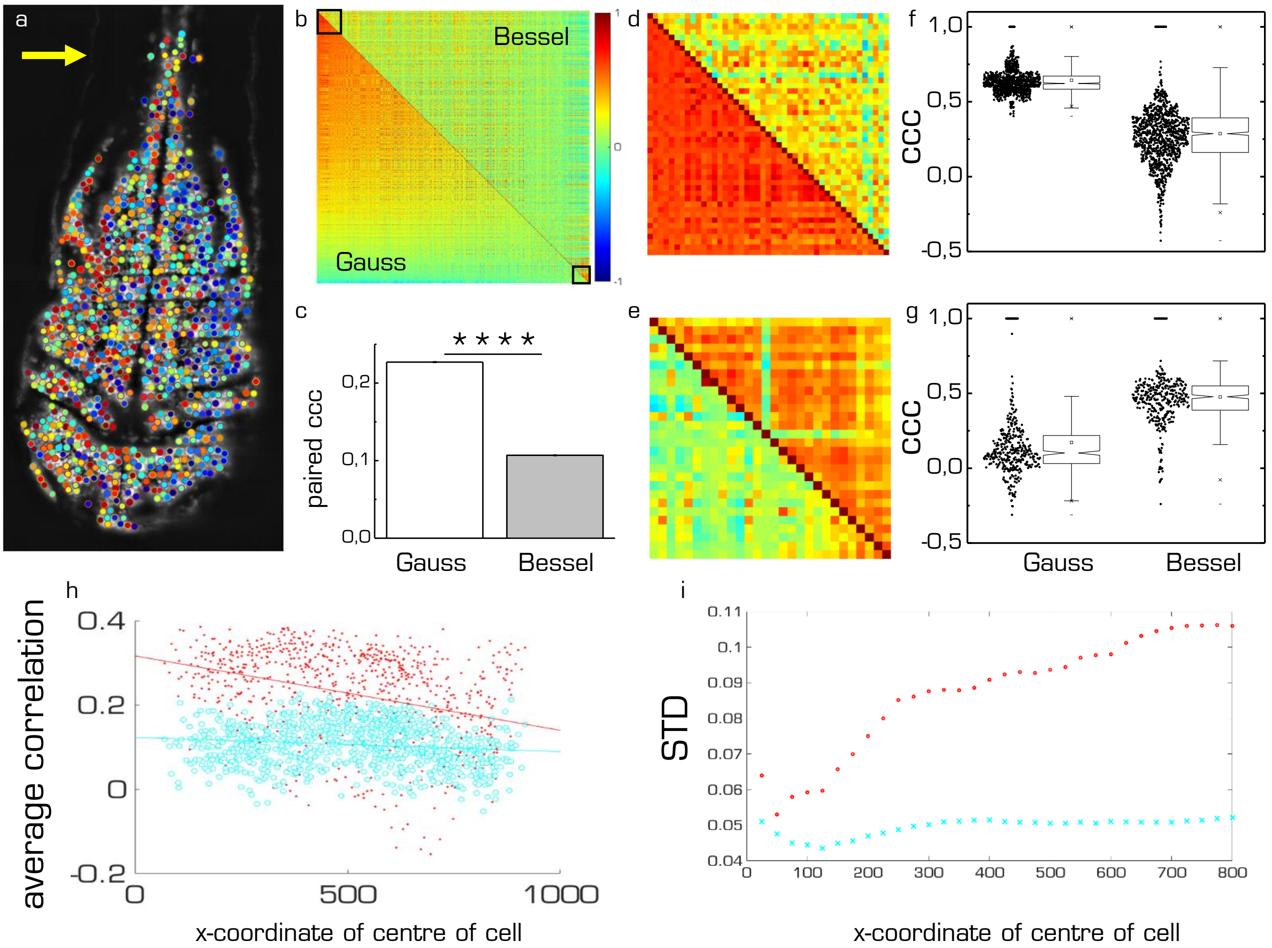

Figure S7. Extension to automated segmentation. **(a)** Automated segmentation (using the script published in Kawashima et al. (2016)) of 807 cells in a transverse plane of a 4dpf elavl3:H2B-GCaMP6s larva imaged with Gaussian illumination. Yellow arrow indicates direction of illumination. After registration of the time lapse imaged with Bessel beam illumination to that imaged with a Gaussian beam, the same coordinates were used to extract the df/f traces from the exact same cells in both imaging modalities. **b** Cross correlation matrix comparing Bessel (upper triangular matrix) and Gaussian illumination (lower triangular matrix) for the 807 cells automatically segmented in a. The cells appear in order of correlation strength in the Gaussian correlation matrix. **c** Average cross correlation coefficient of data shown in b $p < 0.0001$, paired t-test, n=807 cells, in N=1 larva, error is sem. Details of the cells with strongest correlation (**d**) and anticorrelation (**e**) corresponding to the square indicated in b, and the corresponding paired data populations in **f,g** respectively. **h** Average correlation of each cell plotted against the x-coordinate of its center for Gaussian (red) and Bessel beam illumination (cyan). Also indicated are the least-square lines for each distribution. **i** Cumulative standard deviation of the average correlation of each cell for Gaussian (red) and Bessel beam illumination (cyan).

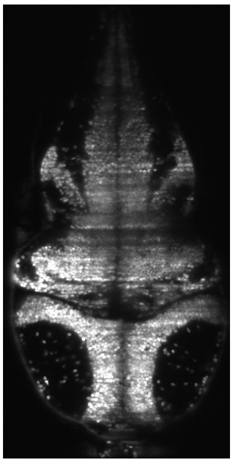

Figure S8. Movie1: Transverse plane of a 5dpf elavl3:H2B-GCaMP6s larva. Raw data time lapse imaged with a Gaussian beam. See Fig.S3 for details.

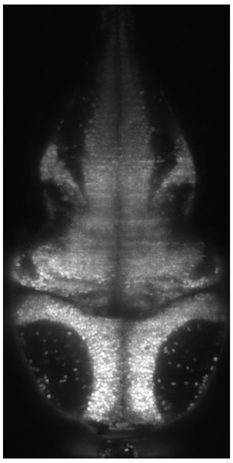

Figure S9. Movie2: Transverse plane of a 5dpf elavl3:H2B-GCaMP6s larva. Raw data time lapse imaged with a Bessel beam. See Fig.S3 for details.

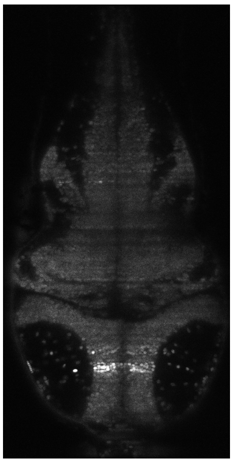

Figure S10. Movie3: Transverse plane of a 5dpf elavl3:H2B-GCaMP6s larva. Standard deviation time lapse imaged with a Gaussian beam. See Fig.S3 for details.

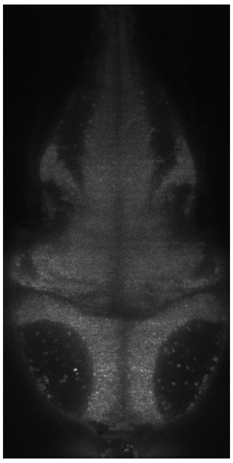

Figure S11. Movie4: Transverse plane of a 5dpf elavl3:H2B-GCaMP6s larva. Standard deviation time lapse imaged with a Bessel beam. See Fig.S3 for details.

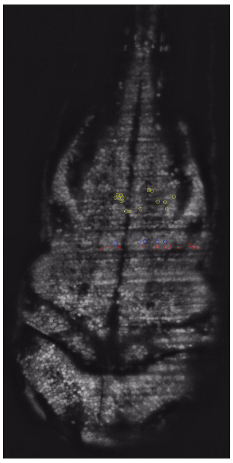

Figure S12. Movie5: Transverse plane of a 4dpf elavl3:H2B-GCaMP6s larva. Time lapse of spontaneous neuronal activity imaged with a Gaussian beam. The raw data was despeckled and local contrast was enhanced with standard FIJI functions.

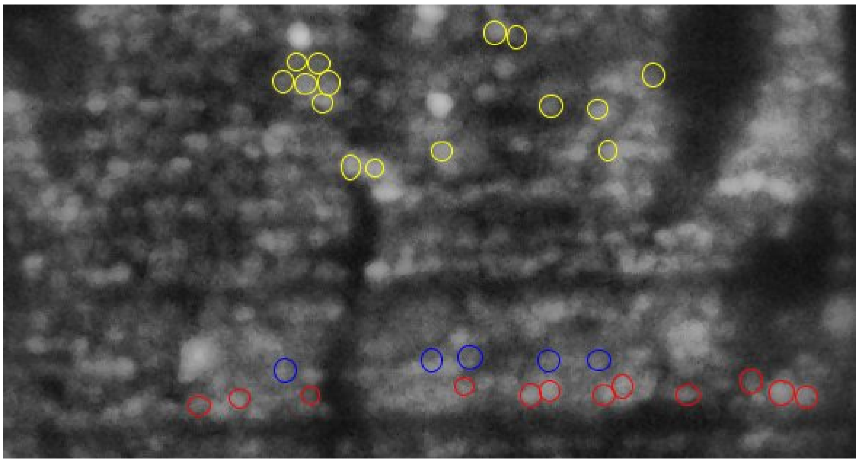

Figure S13. Movie6: Transverse plane of a 4dpf elavl3:H2B-GCaMP6s larva. Time lapse detailing the cells of spontaneous neuronal activity imaged with a Gaussian beam. The raw data was despeckled and local contrast was enhanced with standard FIJI functions.
